# Hate in the time of the Covid-19 pandemic: dehumanisation as a side effect; *re*-humanisation as a remedy

**DOI:** 10.1007/s10611-022-10073-8

**Published:** 2023-01-13

**Authors:** Melanie Collard

**Affiliations:** Department of Law and Criminology, School of Law and Social Sciences, Royal Holloway, University of London, Egham, Surrey, TW20 0EX UK

**Keywords:** Dehumanisation, Racist violence, Hate crime, State crime, Re-humanisation, Academia

## Abstract

This article is about denouncing the dehumanisation process that took place in the time of Covid-19. It recognises that governments have a vital role to play in setting national directions to tackle racist violence and that the value of having hate crime laws should not be underestimated. However, it argues that a broader approach is needed to embark upon a *re*-humanisation initiative and effectively combat racist violence. It emphasises that, to get people truly devoted to a course of action, they must develop a greater understanding of the sources of the problem. Accordingly, this article suggests that academia has a key role to play in shedding light on the occurrence of *de*-humanisation and the potential for *re*-humanisation.

## Introduction

Coronavirus, with its worldwide spread has caused millions of deaths globally and disrupted the day-to-day activities of the whole world. People were facing several problems, be it medicines, masks, sanitizers or food. Throughout the pandemic, many people around the globe extended their helping hands and supported those in need: health and key workers on the frontline; people delivering food to the isolated and vulnerable; designers developing emergency masks and ventilators, etc. Those acts of kindness restored our faith in humanity and reminded us that people have this incredible capacity for cooperation; it is what makes us human in many ways.

And yet we have this capacity for othering. The contagion began towards the end of 2019 in the region of Wuhan in eastern China. Following that, many political leaders and media commentators adopted the practice of calling the ailment the ‘China Virus’, ‘Chinese Flu’ or some other variant that made reference to China or Wuhan, rather than ‘coronavirus’ or ‘Covid-19’, the terms used by the WHO and other health officials.^1^ This discourse pointed those who needed someone to blame—not just an invisible enemy, as ephemeral as a virus (Lerner & Miller, 1978)—to people of Asian heritage, as well as others, and some responded with hateful violence. These incidents, often involving criminal behaviour, are a form of ‘dehumanisation’ or ‘othering’, defined as viewing or treating (a person or group of people) as intrinsically different and alien to oneself (Kelman & Hamilton, 1989). Historically, pandemics have been associated with intensifying fears of ‘the other’ and escalating racist myths about foreigners and discrimination against minorities. Dehumanisation has been a steadily growing social problem since the new millennium: ‘a migration crisis, political upheaval due to populism, disinformation and the pandemic increased feelings of insecurity, and make the future unforeseeable’ (Bayer & Bárd, 2020: 20).

This article is about denouncing the dehumanisation process that can take place in times of change. This article will emphasise that governments have a vital role to play in setting national directions to tackle dehumanisation and that the value of having hate crime laws in place should not be underestimated, particularly at a time when ‘difference’ and ‘otherness’ are coming under increasing scrutiny. Furthermore, it will suggest that a broader approach is needed to prevent dehumanisation. More specifically, this article argues that Academia needs to embark upon a re-humanisation initiative to combat racist violence and hate crime. It recognises that, to get people truly motivated, engaged, and devoted to a course of action, they must develop a greater understanding and appreciation of the sources of the problem. Therefore, this article intends to increase awareness by shedding the light on one specific symptom, that of de-humanisation. It begins with an explanation of the concepts of racist violence and ‘hate crime’. The next section will discuss the occurrence of hate crime and the legal framework pertaining to it in England and Wales. A review of the main literature on dehumanisation will then be provided in an effort to analyse the specificities of the racist violence that has been taking place throughout the Covid-19 pandemic. It will then explore the potential for a re-humanisation initiative.

## Racist violence and/or hate crime?

Despite its seemingly palpable core, there is no agreed definition among researchers about what ‘violence’ means (Brubaker & Laitin, 1998, Hearn, 1998, Stanko, 2003). Violence is an ambiguous and elastic concept (Tilly, 1978: 174), covering the direct use of force to cause physical harm or death, through the compelling or inducing of actions by bullying, verbal abuse, intimidation or threat, to partly or fully metaphorical notions of cultural or symbolic violence (Bourdieu & Wacquant, 1992: 167–174). Since there is no hierarchy of violent harm, the meanings of violence are multiple, complex and often contradictory. In the absence of a standard definition, what violence means is embedded within its context (Ray & Smith, 2001: 205; Stanko, 2003: 3). Therefore, understanding the concept of violence first requires that we specify what has happened, when, where and between whom; only then we can think creatively about breaking violence as a social phenomenon (Stanko, 2003: 11). Domestic, racist, homophobic, and sexualised violence are just a few forms of violence that may best be understood when set in their social, historical, and political contexts. Racist violence will primarily be discussed in this article because it is particularly linked to the side effect of the pandemic.

Racist violence is intimately connected with a wider culture of racism, exclusion and violence, and, as such, is capable of being understood only in this context. As Bowling explains, becoming a victim of racist violence ‘does not occur in an instant or in a physical or ideological vacuum’ but rather within a specific social, political, and historical context (1999: 285). Similarly, Ray and colleagues argue that perpetrators of racist violence are heavily imbued with racist assumptions that have a long history in certain societies and have had particular impact in areas that have suffered marked change (2003: 112). Racist violence has no universal definition in law, nor is there any consensus from sociologists as to what the concept encompasses (Goodey, 2007: 571). Witte defines racist violence as the (threat of) violence in which victims are selected ‘not in their capacities as individuals, but as representatives of imagined minority communities based on phonotypical characteristics, and/or religious, national or cultural origin’ (1996: 11), whereas Bowling and Phillips describe it as, ‘violence specifically targeted against ethnic minority communities and incidents that are aggravated by racism and racial prejudice’ (2002: 108).

Racist violence, like violence, encompasses anything on a continuum–from abusive remarks, broken window and slogans daubed on walls through to serious assault, murder and genocide (Allport, 1954; Bowling & Phillips, 2002: 39; Perry & Alvi, 2012: 57–71). In a similar vein, Goodey notes that racist violence is not only manifested as brutal violent acts against the individual, but ‘emerges in the form of everyday occurrences, which have the potential for violence, and which have a steady and negative impact on vulnerable individuals and communities’ (2007: 571). This continuum, first developed by Kelly (1988) in the context of domestic violence, is further explained by Ray and Smith (2001) with regard to racist violence and will be of great significance in the following developments of this article. Indeed, harassment could be an everyday, arguably minor, manifestation of racism but its connections to more violent, or more ‘serious’, incidents of hate crime are well recognized (Bowling, 1999; Mason, 2005). In this context, one can only emphasise the importance of early intervention to prevent the escalation of hostility (Allport, 1954).

Ray and Smith explain that racist violence typically (though not always) takes place between people who know each other, and is generally embedded in social relations where its use has meaning to victims and perpetrators, especially to keep people ‘in line’ (2001: 205). According to these authors, this is what differentiates racist violence from a classic ‘hate crime’, which they define as ‘stranger violence against interchangeable victims’ who are selected because of their ‘perceived membership of a particular social category, defined for example by ethnicity, gender, nationality, social class, or sexuality, and is unacquainted with his or her attackers’ (Ray & Smith, 2002a, b: 7). The types of prejudice most commonly recognized as coming within the ambit of hate crime are those that are associated with the victim’s ‘minority’ status (or perceived status), such as those based on race, ethnicity, sexuality, colour, religion, disability and gender. It is integral to most definitions of hate crime that the victim is chosen purely on the basis of his or her membership of a particular minority group (Mason, 2005: 839; Khan, 2002) and, consequently, ‘the individual identity of the victim is irrelevant’ (Lawrence, 1999: 9). This, in turn, encourages the assumption that the victims and perpetrators of hate crime tend to be strangers to each other, an assumption reflected in a solid body of empirical research on the topic (Bowling, 1999; Lawrence, 1999; Levin & McDevitt, 2002). But this ‘prototypical image of hate crime’ (Wang, 1999: 802–3) is self-reinforcing to the extent that it determines the kinds of incidents that are accepted as hate crime in various contexts: incidents where the victim and the perpetrator do know each other may be excluded: ‘[T]he conceptualization of hate crime as crime committed *solely* because of the status characteristics of the victim or hatred of a particular group is likely to limit the inclusion of crimes where victim and perpetrator are in some kind of pre-existing relationship when a prejudice-related incident erupts between them (e.g. in the context of a pre-existing dispute between neighbours, which may be perceived by police, and victims, as a personal dispute)’ (Mason, 2005: 839). There is now a small but well-established literature that actively challenges the stranger-danger image of hate crime by arguing that perpetrators may be *more likely than not* to be known to the victim (Clancy et al., 2001; Mason, 2005; Maynard & Read, 1997; Sibbitt, 1997; Stanko, 2001) and that the very ‘logic of the stranger obscures our ability to understand the ordinariness of hate crime’ (Stanko, 2001: 323). Some scholars also suggest that the term ‘bias crime’ more accurately describes incidents motivated by prejudices as it emphasises the offender’s bias, rather than hatred, towards a victim (Lawrence, 1999; Perry, 2006; Iganski, 2008; Wickes et al., 2016).^2^

Notwithstanding these scholarly attempts at conceptualising racist violence and hate crime, ‘academics, policy makers and practitioners continue to grapple with labelling and defining incidents motivated by an offender’s prejudice towards a victim’s identity’ (Wickes et al., 2016: 240). What may or may not constitute a hate crime is widely contested and is ‘discerned by social movements, codified in law, affirmed by the courts, reconstituted at the level of law enforcement policy and practice, and made publicly available by the media’ (Jenness, 2007: 143). At its core, hate crime is a social construct that is culturally and historically situated (Perry, 2001; Williams & Tregidga, 2014), ‘resulting in a wide array of legal definitions across countries and jurisdictions’ (Wickes et al., 2016: 240). Ultimately, ‘official classifications are shaped by the interpretation of the victim and the criminal justice system, and not academia’ (Chakraborti & Garland, 2009: 150). ‘Hate’, as determined by criminal justice system legislation, is evident in only a small number of hate crimes (McDevitt et al., 2010). Indeed leading scholars in the field argue that official definitions of hate crime are not only highly restrictive but unnecessarily exclude a range of incidents (Chakraborti & Garland, 2015; Hall, 2005, 2015). Adopting a definition that enables inclusive approaches that leverage higher levels of criminal justice engagement to mobilize the criminal law in response to what is usually referred to as hate incidents is key, however, ‘terminology is one of the key factors that often gets in the way of effective policing practice’ (Wickes et al., 2016: 239). For instance, Chakraborti and Garland suggest that ‘conceiving of hate crime through the lens of perceived vulnerability and “difference” gives effect to the realities of targeted victimization, and in so doing allows us to transcend the homogenized generalizations all too prevalent within scholarly and policy domains’ (2012: 510). Further research will provide evidence of which term is more appropriate to describe the bias-targeted violence that has been taking place during the Covid-19 pandemic.

## Hate crime: occurrence & legal framework in England and Wales

The Covid-19 pandemic has led to a raft of unprecedented legal and regulatory ramifications for the UK and other nations. However, unlike other novel components of the coronavirus crisis, hate crime has been firmly on the legal reform agenda for some time. Broadly speaking, hate crime laws that have developed in recent decades have taken two main forms: (1) the creation of specific offences of hate crime or a harsher sentencing regime, where it can be shown that the offence was related to the victim’s membership or presumed membership of a particular group; and (2) the creation of hate speech offences, criminalising incitement of hatred. Some jurisdictions have opted for one or the other of these measures, whilst others, such as England and Wales, have a combination of the two. Indeed, at present, there are a number of different legal provisions that comprise hate crime in England and Wales which encompass five protected characteristics: race, religion, sexual orientation, disability and transgender status. The three main forms of hate crime laws relate to (1) aggravated offences (Crime and Disorder Act 1998); (2) enhanced sentencing (Criminal Justice Act 2003, and now in the Sentencing Code via the Sentencing Act 2020); and (3) ‘stirring up hatred’ offences (Public Order Act 1986).

Consequently, the defining feature of hate crime laws in England and Wales, as in many other jurisdictions, is sentence enhancement through either specific aggravated versions of some existing criminal offences (Crime and Disorder Act 1998), which allow for a higher maximum sentence for each of the underlying offences, or an enhanced sentencing regime (Criminal Justice Act 2003), which applies to all other offences and requires an increase in penalty within the existing maximum for the base offence (Chalmers & Leverick, 2017; Law Commission, 2020). However, any legal punishment of wrongdoers by the state must be justified because it inflicts deliberate suffering on individuals and infringes a person’s liberty and autonomy (Ashworth, 2009: 22; Schonsheck, 1994: 1; Raz, 1986: 416; Dolinko, 2011: 403). Similar logic has been applied to more severe punishment, which amplifies these effects. As Cavadino argues, ‘[J]ustification for enhanced punishment needs to be sought within the context of whatever justification there may be for punishment more generally’ (2014: 1). There are a number arguments that justify punishing hate crimes more severely than differently motivated crimes: (1) hate crime causes additional harm, namely to primary victims, but also to groups who share the targeted characteristic and to society more widely; (2) hate crime constitutes greater intrinsic wrongdoing; (3) hate crime offenders are more culpable than those who commit equivalent offences which are not hate crimes; and (4) more severe punishment sends out a message, denouncing the hatred as wrong (Law Commission, 2020: 55; Chakraborti & Garland, 2014: 1; Lawrence, 1999: 169; Walters, 2014: 47–74). The first three arguments suggest that hate crimes are more serious than differently motivated crimes because they convey greater harm, greater wrongdoing, and greater culpability and, therefore, greater punishment might be justified according to retributivist/’just deserts’ theories of punishment. Hate offences have a considerably greater impact than ordinary crimes on direct victims, the victim’s community and society as a whole (Hardy & Chakraborti, 2016). Since the victims of hate crimes are often targeted for an immutable, unchangeable characteristic, or one that is the core of one’s identity, the impact of the crime, the feeling of vulnerability, helplessness and hopelessness on the side of the direct victim may be especially grave (Iganski, 2001; Iganski & Lagou, 2014). In that sense, an offence motivated by hatred of the victim’s very identity causes *additional harm* to the victim (and to the victim’s group) than if it was not so motivated because it diminishes them, denies them dignity, and may make them doubt their safety and acceptance in society (Law Commission, 2020: 55–57). In the 2019/20 Crime Survey of England and Wales, for example, 36% of hate crime victims said they were “very much” affected emotionally after the incident, compared with 15% for all crime victims (Home Office, 2020: 19). And academic studies have concluded that victims of hate crime are more likely than victims of comparable, otherwise motivated crimes to report suffering panic attacks, depression, fear, feeling vulnerable or being in shock (Iganski & Spiridoula, 2015: 34–46; Law Commission, 2020: 55–57). Additionally, the Sussex Hate Crime Project exposed the indirect impacts of hate crimes—that is, how hate attacks on members of a community affect the thoughts, emotions and behaviours of other members of that community (Paterson et al., 2018). Indeed, hate offences are said to be ‘message offences’, in the sense that they may well erode societal cohesion, reinforce social tensions, and trigger retaliation that results in a vicious circle of violence and counter-violence (Burney & Rose, 2002: ix; Iganski, 2002: 135; Perry, 2014; Walters et al., 2018). The fourth argument suggest that denouncing hate crime via more severe punishment could deter existing hate crime offenders and future offenders (specific and general deterrence) as well as ‘morally educate the public conscience’ (Cavadino, 2014: 8), and, therefore, greater punishment might be justified by utilitarian theories of punishment (Dixon & Gadd, 2006: 310). It is beyond the scope of this article to discuss the respective merits of retributivist and utilitarian theories and the extent to which each might justify legal punishment but there is extensive and competing literature available (see, for instance, Easton & Piper, 2016; Dolinko, 2011).

These considerations offer good enough reasons for addressing hate crimes differently than ordinary crimes. Having said that, the scope and implementation of hate crime and hate speech laws is not without its troubles. The legal framework has been described as a “hierarchy of hate” as it does not treat all of the protected characteristics equally (Law Commission, 2020; Walters et al., 2018). However, at the end of 2021 and following a wide-ranging review of hate crime laws, the Law Commission announced recommendations to reform hate crime legislation to ensure that disabled and LGBT + victims receive the same protections as victims with other protected characteristics, such as race and religion (Law Commission, 2021).^3^ If enacted, the reforms would ensure all five characteristics are protected equally by the law. On the implementation front, empirical studies highlight that the current legislative framework for hate crime in England and Wales is overly complex, confusing, and of unequal application (Burney & Rose, 2002; Dixon & Gadd, 2006; Walters et al., 2018). Research has also clearly established that the hate element of a crime can be “disappeared” over its lifecycle and fall into the “justice gap” (Schweppe et al., 2018; Walters et al., 2018). Perhaps Walters and colleagues’ recommendation regarding the enactment of a new “Hate Crime Act” to consolidate the current framework of offences and sentencing provisions could offer much-needed simplicity to the comprehension of the law, as could their proposition to implement a new “by reason” test, replacing the “motivation of hostility” test set out in the current legislation (Walters et al., 2018: 31–32).^4^ Furthermore, Iganski (1999) is sceptical about the likely deterrent effects of such marginal increases in the severity of sentences on either the individuals who receive them, or generally on the public at large, for reasons to do with the theory and practice of specific and general deterrence (Ashworth, 1998). Also, Bowling and Phillips (2002) consider the fact that a more severe punishment for these offences could lead to further victimisation of minority communities. In the same vein, Dixon and Gadd (2006) point out that the confidence of minority ethnic groups in the law may be undermined if anti-hate crime legislation is used too readily against them. Finally, some authors have argued that the further criminalisation of already disadvantaged and marginalised people is too high a price to pay for creating the impression that the criminal justice system is taking ‘race equality’ seriously, particularly in the absence of convincing evidence that the incidence of hate crime has fallen since hate crime and hate speech laws came into force (Burney & Rose, 2002; Dixon & Gadd, 2006; Ray et al., 2002, 2003, 2004).

Obviously, the legislation is only a small part of the solution to any substantial societal ill like hate crime. Indeed, events such as the Brexit referendum, the terrorist attacks in London and Manchester and the Covid-19 pandemic offer ample evidence of just how sizeable a problem hate crime continues to pose to communities and groups of victims. The latest Home Office figures have highlighted the scale of racist violence. In 2020/21, there were 124,091 hate crimes recorded by the police in England and Wales, an increase of 9% compared with 2019/2020 (Home Office, 2020, 2021). The majority of hate crimes were racially motivated, amounting for 74% of such offences, an increase of 12% between year ending March 2020 and year ending March, 2021 (Home Office, 2020, 2021). Furthermore, in June 2020, at the height of the Black Lives Matter protests and far-right groups counter-protests following the death of George Floyd on the 25 May in the United States of America, racially or religiously aggravated offences in England and Wales were a third higher (34%) than they had been in June 2019, continuing the upward trend in these offences seen in recent years (Home Office, 2020: 30–32). Whether this results from increased recording, increased reporting or increased violence remains an open question.^5^

The persistence, or even ‘spikes’, in hate crime perpetration confirm that the laws alone are not enough to foster tolerance and understanding within society, or to prevent disturbingly high levels of hate crimes from being committed. Indeed, Ray and Smith (2002a, b) explain that the legislation is not the most appropriate tool since localised cultures of racism and violence tacitly support such behaviour. Consequently, an adequate account of the way racist violence is experienced and understood by the individuals involved—perpetrators as well as victims—and by other people living in the neighbourhoods from which they come need to be taken into account.

## The dehumanisation process: a state crime?

Modern psychology and behavioural sciences demonstrate that ordinary people are capable of great kindness, just as they are also capable of great cruelty (Clarke, 2008; Milgram, 1963, 1974; Haney et al., 1973). This may cast doubt on the ‘few bad apples’ theory. A great deal depends on the facts of a given situation, which can bring out the best—or worst—in everyone. Those who emphasise the relative or absolute importance of social and environmental factors over individual psychological characteristics invariably point to the studies by Milgram (1963, 1974) of obedience to authority, and those by Haney et al. (1973) and Zimbardo (2007) of the structure of bureaucracy. Both their experiments provide empirical evidence that, even when they have been socialised for many years against the infliction of cruelty and dehumanisation, ordinary people can regularly and reliably carry out violence against another human being. These two studies were prompted by claims in the aftermath of WWII that those working in the death camps and perpetrating genocide were simply following orders. Scholars set out to test whether ordinary people could be induced to engage in cruel exercises by means of authoritative commands. The combined results of both experiments suggest that situational power can induce the vast majority of ‘normal’ people to engage in what can only be described as the dehumanisation of other people (Huggins et al., 2002: 263; Clarke, 2008: 12; Osiel, 2004: 135). From these experiments, it became clear that it’s extremely easy to turn down someone’s ability to see someone else in their full humanity. Dehumanisation, defined as the gradual and progressive exclusion of members of certain groups, achieved by depriving them of their humanity and redefining them as enemies (Kelman & Hamilton, 1989), is a prime way of legitimising, in the eyes of the perpetrators, the moral transgressions against the people who have been scapegoated (Crelinsten, 2003: 301; Fein, 1990; Lankford, 2009; Zimbardo, 2007). In this manner, dehumanisation of the ‘other’ is a psychological loophole that allows offenders to engage in hate crimes. These experiments, if anything, probably understate the level of ‘othering’ that could take place in a time of pandemic.

No society is immune from the signs of dehumanisation, but whether they get tamed or deepened, depends on the social measures that are applied vis-à-vis the phenomenon: ‘Whether by speech, action or omission, the state’s reaction creates norms, and informs society about the current acceptable standards’ (Bayer & Bárd, 2020: 13). States can play an important role in dehumanisation (Kelman, 2005: 133). In colonial settings, for instance, racist ideologies were used to cast the colonised person as ‘other’ (Fanon, 1963; MacMaster, 2004; Vidal-Naquet, 1963). The ‘others’ were consequently treated as guilty non-citizens who were not entitled to government protection, and as people ‘who have placed themselves outside the moral community shared by the rest of the population’ (Kelman, 2005: 133). Once the ‘others’ are placed outside the perpetrators’ moral universe, there is no normal human obligation towards them (Fein, 1990). One way to achieve this is the use of certain terms to refer to the ‘others’. Indeed, the perversion of language plays an important role in the dehumanisation process; it helps to make acts of violence easier for the perpetrator. The use of such vocabulary enables perpetrators to ‘construct a social reality that distorts facts and events, reallocating the infliction of pain in a different cognitive world’ (Cohen, 2001: 83). This socially constructed reality supplants conventional morality, substituting in its place the ideological dictates of the authority structure within which hate crime occurs. Looking back at some of the most tragic episodes in human history, we find words and images that stripped people of their basic human traits: Nazis depicted the Jews as ‘rats’, ‘vermin’, characterised by ‘vanity’, ‘soullessness’, ‘stupidity’, ‘malice’, etc. (Browning, 1998); the U.S. military encouraged soldiers to call Iraqi prisoners ‘dogs’ (Lankford, 2009); in Rwanda, Hutu officials called Tutsis ‘cockroaches’ that needed to be cleared out (Kassimeris, 2006: 9); and in Algeria, tortured victims who were dumped at sea were given the derisive name of ‘shrimps’ (Vidal-Naquet, 2001: 72).

Dehumanisation, however, does not only occur in wartime. As Bourke notes, ‘it is not necessary to look for extraordinary personality traits or even extraordinary times to explain human viciousness. Numerous studies of cruelty show how men and women *like us* are capable of grotesque acts of violence against fellow human beings’ (1999: 5). More specifically, states not only seem to fail in addressing dehumanisation, but some of them are instigating it. As the UN Special Rapporteur on contemporary forms of racism, racial discrimination, xenophobia and related intolerance has put it, ‘political rhetoric, especially nationalist populist ideologies pose a threat to equality by fuelling discrimination and intolerance’ (United Nations General Assembly, 2018). The impact of this type of rhetoric was aggravated by and became more visible during the Covid-19 pandemic (Bárd & Carrera, 2020). Indeed, referring to the coronavirus as the ‘Chinese flu’ led to incidents targeted at minorities,^6^ especially people who were taken for having an Asian background, in the US, UK and elsewhere in early 2020: in New York, an Asian passenger was sprayed with Febreze and verbally abused on the subway; in Poland teenagers attacked, threw garbage, and spat at a Vietnamese woman, while shouting racist slurs connecting the victim’s origin to the virus; in London, a twenty-three-old Singaporean was brutally attacked while walking on busy Oxford Street by a man who shouted ‘I do not want your coronavirus in my country’; in Estonia, a person of Malaysian origin was shouted at and blamed for bringing the virus into the country; in Texas, three Asian American family members, including a two-year-old and six-year-old, were stabbed by a man who claimed he attacked them because he thought the family was Chinese, and infecting people with the coronavirus; in Exeter, three victims from Chinese and Asian backgrounds were punched, kicked, spat on and told to ‘go back to your own country, you must have coronavirus’ (BBC News, 2020; ENAR:, 2020; Miebai, 2020; Russell, 2020; Wu, 2020). These are only a few examples of incidents that have been reported and recorded.

The hashtag movement #JeNeSuisPasUnVirus (I am not a virus) reportedly starting in France and spreading across Europe and beyond, reflects the increasing frustration of minority citizens, who are the victims of the proliferation of prejudice, hate speech and hate crimes (Coste & Amiel, 2020). Similarly, as coronavirus fears continued to mount, Twitter expanded its ban on ‘dehumanising language’ to include disease. The Twitter safety team explained that a rule barring hate speech targeting religious groups now applies to ‘language that dehumanises on the basis of age, disability, or disease’, basing their argument on research which shows that dehumanising language increases the risks of offline harm (Panache, 2020). Examples of rule-breaking tweets included posts that refer to people with a disease as ‘rats that contaminate everyone around them’ or ‘subhuman’ (Panache, 2020).

Scapegoating during a heath crisis is nothing new. Historically medical doctors and nurses were blamed for being incapable of stopping the plague (Bayer & Bárd, 2020: 17). During the medieval plague, pogroms were organized, based on the conspiracy theory that the disease was deliberately spread by Jews through well poisoning (Williamson, 2020). Xenophobia and racial prejudice have been associated with infectious disease outbreaks in Europe and in Asia in the 1500s, when each affected country blamed their neighbouring countries or enemies for their outbreak (Ng, 2020). Similarly, it has often been claimed that the Gypsy community presents a threat to public health (Holt, 2020: 659). Exhibition such as ‘Germ City: Microbes and the Metropolis’ at the Museum of the City of New York or the Wellcome Trust ‘Contagious Cities’ project across Berlin, Geneva, Hong Kong and New York have all emphasised that, throughout history, pandemics had often intensified discrimination and crimes against minorities and immigrants who were seen as carrying yellow fever, trachoma, or SARS (Russell, 2020). It therefore came as little surprise that the Leeds-based organisation Stop Hate UK observed a spike in hate crimes and incidents reported by Asian communities and individuals in the UK at the beginning of the Covid-19 pandemic (Russell, 2020).

Not only did the coronavirus take human lives, but it reinforced existing problems, and hit harder on otherwise vulnerable minorities. During the outbreak of Covid-19, states derogated from constitutional checks, and limited rights and freedoms of their citizens, residents and foreigners. In this climate hostile towards democracy, dangerous with respect to the rule of law, human rights are also more prone to be infringed: ‘A pandemic does not turn state agents and societies into human rights violators, but it shows more clearly their true colours, i.e. pre-existing problems and social tensions […] Covid-19 is no exception’ (Bayer & Bárd, 2020: 16). What are the origins of this pre-existing ‘racist sentiment’ then? Smith argues that it is a sentiment that ‘infuses daily life and is widely but abstractly expressed by a broad cross-section (perhaps a majority) of the population’, a ‘reservoir of procedural norms that not only tacitly inform routine activity, but are also able to legitimise more purposive explicitly racist practices’ (1989: 149–150). The history of race and Britain, for instance, can be traced back to the philosophies of the European Enlightenment and to the ascendancy of science and reason within the work of writers such as Hume, Kant and Hegel. According to them, the acquisition and rule of far-away territories, and the ensuing subjugation of colonial subjects described as ‘being akin to children or animals, needing care, nurture and improvement under a civilised and beneficent rule’ (Bowling & Phillips, 2002: 3), was reflective of the biological basis for racial supremacy during this period of empire building, and was central to the emergence in the mid-nineteenth century of the scientific discourse of Social Darwinism and its emphasis on the superiority and preservation of the European white race. However, Chakraborti and Garland (2009: 20–21) note that it is the post-Second World War period that arguably has the most significant implications for the way in which the recent British ‘race’ agenda has developed. Indeed, the post-war influx of migrants into the UK—which occurred primarily as a way of filling labour shortages—quickly resulted in a noticeable different demographic profile. Even though this immigration was not confined solely to incomers from the Caribbean and Asia, it is the migration of inhabitants of New Commonwealth countries that appears to have shaped subsequent debates and policy-making on race relations: the Commonwealth Immigrants Act 1962, the notorious ‘rivers of blood’ speech of conservative MP Enoch Powell in 1968, the Commonwealth Immigrants Act 1968, the Immigration Act 1971, and the British Nationality Act 1981 of Thatcher, are but a few example of the continuing racialisation of immigration policy on the lines of skin colour.

It is widely accepted that racist violence cannot be seen only as the madness of individual hooligans or simply as ‘politically motivated’ (Ray et al., 2000: 29). Racist violence ‘is a much broader phenomenon that includes violence perpetrated by individuals and groups of youth in contexts where the implicit *goals* are shared among a much broader section of the English population’ (Bowling & Phillips, 2002: 125). While there clearly are committed racist offenders—since violence can be ‘targeted’ against a minority ethnic group (Stanko: 2001)—the motivation underlying the majority of what ends up being classified as a ‘racist incident’ is often ambiguous. Indeed, this feeling of shame resulting from a supposedly ‘improper’ relationship between whites and ethnic minorities has its origin in the taken-for-granted everyday racism of offenders’ communities, something Iganski refers to as the ‘normality of everyday hate crime’ (2008: Chap. 2). Drawing on criminological theory and research evidence, it appears that the explanations might be ‘social rather than individualistic’ ones (Ray & Smith, 2004: 686)—an idea more in line with Beck and Tolnay’s (1995) integrated theory, which explains racist violence as product of individual racism as well as of a certain permissiveness of the state.

It is this permissiveness of the state that allows to conclude that certain hate crimes, including those that have taken place following the Covid-19 pandemic, could be labelled as state crimes in the sense that states have failed to develop and implement robust responses to these forms of offences and, as such, have entered the realm of criminal omission. International law—through, for example, the 1948 Universal Declaration of Human Rights in 1948 and the 1966 International Covenant on Civil and Political Rights at the global level and the 1950 European Convention on Human Rights^7^ at the regional level—provides guidance on how to prevent and tackle the phenomena, but it is ultimately up to the states to implement measures that efficiently combat this issue. As explained by Schweppe and colleagues, ‘the very presence of an official statement from Government that hate crime, hate speech, and any form of discrimination are abhorrent in a modern democracy and contrary to human rights is vital to ensuring that the “message” that hate crime is unacceptable is communicated clearly and unambiguously’ (Schweppe et al., 2018: 52). This ‘hate crime/state crime’ claim is linked to (a) the doctrinal principle that an omission to act (such as a failure to provide appropriate protection) could lead to criminal liability; and (b) the positive state obligations that can be derived from the substantive provisions of human rights conventions if they are read in conjunction with the general principle that everyone’s rights and freedoms within the contracting states’ jurisdiction have to be secured (more specifically the right to life, freedom from torture). In line with Barak and Bohm’s (1989) argument, this article emphasises that the existence of widespread hate crime is not only an appropriate subject of criminological investigation but also a social problem that needs to be attributed to state criminality. While relying solely on a human rights discourse would leave us with the borderless condition of ‘social harm’ which is less satisfactory for a criminological perspective, the concept of criminal omission could provide an appropriate framework. The doctrinal principles of *actus reus* and *mens rea* could be used to argue that we are not just dealing with ‘non-state hate crimes and what states should do about them’ but an (intentional or reckless) criminal omission to prevent fatal and non-fatal offences against the person by failing to provide adequate protection and, consequently, failing to inform society about the current acceptable standards. Whilst the law is an important tool that States can use to expressly denounce prejudice-based conduct (Schweppe et al., 2018: 53), it is by no means the only one: ‘The problem of hate speech and hate crime should be regarded as a complex social problem, and a symptomatic response given by societies to the challenges which have not been adequately managed’ (Bayer & Bárd, 2020: 21). Therefore, an adequate state response should be similarly complex: addressing the underlying issues and the symptoms at the same time.

## From *de*-humanisation to *re*-humanisation

In late March 2020, U.N. Secretary-General Antonio Guterres said the world is not only fighting the ‘common enemy’ of the coronavirus ‘but our enemy is also the growing surge of misinformation’ about Covid-19 disease. To overcome the virus, Guterres said that ‘we need to urgently promote facts and science and promote hope and solidarity over despair and division,’ and he urged all nations ‘to stand up against the increase in hate crimes targeting individuals and groups perceived to be associated with the coronavirus’ (Lederer, 2020). Miguel Moratinos, head of the U.N. Alliance of Civilizations, and Adama Dieng, the U.N. special adviser on the prevention of genocide, also expressed grave concern at the increase in stigma, hate speech and hate crimes over the pandemic: ‘We are all facing the same enemy, one which is invisible, rapidly advancing, taking lives away and causing havoc indiscriminately […] but allowing it to tear apart the fabric of our societies is perhaps one of the most serious upheavals that the Covid-19 pandemic is inflicting upon our world’ (Lederer, 2020).

So how could states counteract the symptom of dehumanisation more effectively? According to Bayer and Bárd, ‘A humanistic and rational state policy is capable of curbing revenge, cruelty, brutal instincts, and aggression resulting from a lack of knowledge’ (2020: 20). States should promote the effective collaboration of law-makers, law-enforcers, non-governmental organisations and activists to develop and implement robust responses to these forms of offences. There is a shared responsibility in a system of multi-level governance to tackle the issue of hate speech and hate crimes, an area on the border between human rights law and criminal justice. Governments have a vital role to play in setting a national direction to tackle dehumanisation and the value of having hate crime laws in place should not be underestimated, particularly at a time when ‘difference’ and ‘otherness’ are coming under increasing scrutiny. Legal prohibition is and remains one among the important symbolic messages and actions with which a state can express its values and set its standards (Bayer & Bárd, 2020: 21). However, a broader *re*-humanisation initiative, involving policies promoting educational approaches, is needed to tackle prejudice and discrimination. In this context, re-humanisation is seen as the process by which society can counteract the damage done by dehumanisation—that is, rehabilitating one’s way of perceiving the other(s) in question in one’s mind and in consequent behaviour.

Academia could therefore play a key role in challenging beliefs, attitudes and harmful narratives before they develop into hatred to generate a healthier community and an atmosphere where hate crimes are less likely to occur. It could aid in creating communities respectful of tolerance and diversity. Indeed, to get people truly motivated, engaged, and devoted to a course of action, they must develop a greater understanding and appreciation of the sources of the problem (Livingston, 2021: xi-xii). An academic agenda of re-humanisation could be built on three pillars: (1) research and further development of in-depth understanding of the changing nature of hate crime; (2) outreach activities to raise awareness regarding the context surrounding bias incidents in order to build skills in identifying, dealing with, and preventing hate crime within the wider society (including criminal justice professionals, schools administrators, students, business leaders); (3) the promotion of responsiveness by clearly informing potential victims and bystanders of the different mechanisms available to report the hate incidents they witnessed.

This is an ambitious endeavour, to say the least, but not one that is marked by blind optimism. In a psychological study conducted at Princeton University, Wheeler and Fiske (2005) showed that re-humanisation can be reached by blocking brain activation which is usually suitable with the appearance of an individual. By helping subjects to unconsciously suppressing the activation of ‘acute stress responses’, re-humanisation was achieved when a person was seen not as a category, racial or other, but as an individual. Here the researchers ‘primed’ the subjects by asking them to guess whether the person whose face they were about to see liked coffee or tea, etc. Although the findings of controlled social-psychological laboratory experiments can never fully portray the realities of real-world violence settings, they can offer parallels that highlight the operation of relevant dynamic process (Huggins et al., 2002: 141). This ‘re-humanising’ experiment, if anything, probably understate the potential of society at large. To build social resilience, notice should be taken of the fears and concerns that make people susceptible to populistic, discriminative or even racist views. Criminological, sociological, linguistic and psychological research could greatly contribute to yielding fresh knowledge about the intriguing success of hate speech and populism: ‘Research to process and decode the “hate narrative” and to define what is the real concern behind hate should be supported by the civil society as a whole’ (Bayer & Bárd, 2020: 13).

It has been argued that hate behaviours serve to reinforce structural boundaries in society that sustain privilege according to race, gender and sexuality because human subjectivity is informed by hegemonic norms (James & McBride, 2018; James, 2020: 2, 6; Perry, 2001, 2006; Messerschmidt’s, 1997). Therefore, the pursuit of anti-racist futures must involve the ‘identification and obliteration of deeply embedded epistemic hegemonies, which have been created through the twin processes of capital expansion and colonisation’ (Dawson, 2020: 75). And if academia is to offer a varied and useful set of perspectives to understand and expose the complexities of the dehumanisation process, then it should ‘revise its histories too’ (Aliverti et al., 2021: 300). This article attempts to play a small part in the re-humanisation initiative by increasing awareness of the issue of racist violence by shedding the light on one specific process, that of de-humanisation.

## Data Availability

N/A.

## Footnotes

1 This was the case of former US President Donald Trump, who attempted to defend his language, explaining that it is simply a way of reminding people from where the virus emanated. He also denied the term is racist or that the term maligns people of Asian heritage (Wu, 2020).

2 It is useful to note that our understanding of the ‘victim-offender relationship’ is often based on police official records where the type of relationship identified is limited and prescribed by the data collection forms used by the police.

3 Between 2019 and 2021, the Law Commission undertook a wide-ranging review of hate crime laws—that is, how hate crime laws work, who they protect, and how they could be reformed to work more effectively). In September 2020, the Law Commission published its consultation paper, a 544-page document, with proposals for reforming the law relating to hate crime to ensure it works effectively (Law Commission, 2020). The Law Commission wanted to hear the public’s views on the proposals, with the consultation ending on Christmas Eve 2020. Key considerations related to the different treatment of protected characteristics, as well as the structure of the hate crime-focused laws and how they fit together. Throughout this period, the Law Commission met with hundreds of stakeholders to discuss their provisional proposals and received 2,473 written responses, a high proportion of which were from individual members of the public (Law Commission, 2021: 8). A significant majority of these personal responses opposed most of the Law Commission proposals for reform, indicating strong objections to hate crime laws altogether or any extension of those that currently exist (Law Commission, 2021: 8). On the other hand, the majority of the 173 responses received on behalf of organisations were supportive of the broad direction of the Law Commission proposals, and in particular the emphasis on parity of protection amongst the existing five characteristics recognised under hate crime laws (Law Commission, 2021: 8). This lack of community consensus for hate crime laws informed the more limited approach the Law Commission has taken to extension of existing laws than it initially contemplated in the consultation paper (Law Commission, 2021: 8).

4 At the time this article was written, the law stayed unchanged.

5 It is risky to rely on the official statistics of recorded racially motivated crimes given that not all racist incidents are reported, and that not all racist allegations reported to the police are recorded as such (Bowling & Phillips, 2002; Bowling, 1999; Chahal & Julienne, 1999; Maynard & Read, 1997, Sibbitt, 1997). Similarly, local crime surveys are not without problems either. For instance, the Crime Survey for England and Wales does not take into account ‘low level incidents’ and so underestimates the extent of racist violence (Brimicombe et al., 2001). What emerges is that the relationship between police records and victimisation surveys remains a complex one, and therefore establishing the extent of racist violence is not an easy task. Nonetheless, police records and victimisation surveys are the two most common methods of quantifying the extent of violent racism.

6 People anyway suffering more from the pandemics than the average person, due to poverty, overcrowded accommodation, the lack of hygienic conditions, lack of equipment for distance learning, or domestic violence – such as ethnic minorities, the LGBT + community, prisoners, migrants, refugees, but also women and elderly people – were further victimised by hate speech and crimes (Bayer & Bárd, 2020: 18; Holt, 2020: 659; ICAR, 2004).

7 The ECHR guarantees freedom of expression in Article 10, including for speech acts which ‘offend, shock or disturb’ (ECtHR, *Handyside v. the UK*, Application no.: 5493/72, 7 December 1976), by also defining its limits. Hate speech is generally not protected by the European Court of Human Rights, and applications can be rejected with reference to Article 17, a classic abuse-of-rights prohibition (ECtHR, *Seurot v. France*, Application no.: 57,383/00, 18 May, 2004). At the Strasbourg level, the relevant cases dealing with the repression of hate crimes effect mainly Article 2 on the right to life, Article 3 on the prohibition of torture and inhuman or degrading treatment and Article 8 on privacy, beyond Article 14 on the prohibition of discrimination. These articles trigger both negative but also positive obligations on states, i.e. they must not only refrain from interference, but they have the ‘obligation to do something’ (Akandji-Kombe, 2007). Positive state obligations can be derived from the substantive provision if they are read in conjunction with Article 1 of the ECHR to have everyone’s rights and freedoms within the contracting states’ jurisdiction secured. This reading implies that ‘States Parties are answerable for any violation of the protected rights and freedoms of anyone within their “jurisdiction” –or competence–at the time of the violation’ (ECtHR, *Assanidze v. Georgia*, Application no.: 71,503/01, 8 April, 2004).
